# Effectiveness and Safety of Botulinum Toxin Type A in the Treatment of Androgenetic Alopecia

**DOI:** 10.1155/2020/1501893

**Published:** 2020-08-04

**Authors:** Yaguang Zhou, Shui Yu, Jimin Zhao, Xinyue Feng, Meinan Zhang, Zigang Zhao

**Affiliations:** Department of Dermatology, Hainan Hospital of PLA General Hospital, Sanya, Hainan Province, China

## Abstract

**Background:**

Androgenetic alopecia (AGA) represents the most frequent clinical complaint encountered by dermatologists and is characterized by a progressive miniaturization of the hair follicle. However, the efficacy and safety of current medical treatment remain limited, and more personalized therapeutic approaches for AGA are needed. Therefore, the present study is aimed at investigating the efficacy and safety of botulinum toxin type A (BTA) in patients with AGA.

**Methods:**

63 patients with AGA meeting the inclusion criteria were included in this study and treated with BTA injection or BTA injection combined with oral finasteride (FNS). In the scalp, 30 sites were injected with 100 U of BTA in each site and patients received BTA after every 3 months for a total of 4 times. Hair counts, head photographs, evaluation scores, and self-assessment were assessed in patients with AGA.

**Results:**

Hair counts in both groups at all time points were significantly higher as compared with those before treatment. After 4 times of treatment, hair counts in the BTA+FNS group were higher than those in the BTA group. Hair growth and density were significantly augmented, and the area of hair loss was attenuated after each treatment as revealed by head photographs. The effective rates of BTA and BTA+FNS groups were 73.3% and 84.8%, respectively, following 4 times treatment.

**Conclusion:**

BTA is a safe and effective therapeutic strategy for the treatment of AGA without adverse effects, and BTA combined with FNS exhibited a superior therapeutic effect than BTA alone.

## 1. Introduction

Hair loss represents the most frequent and distressing clinical complaint encountered by dermatologists in clinical practice. Androgenetic alopecia (AGA), telogen effluvium, and alopecia areata are the three most common types of hair loss, with AGA being the most prevalent type in dermatology practice. AGA or pattern baldness, characterized by a progressive miniaturization of the hair follicle, is a nonscarring hair loss disorder that predominantly affects up to 80% of men and 50% of women during adolescence and postadolescence [1–3]. The prevalence varies across ethnic groups, with higher prevalence rates reported in Caucasian men (50%) and women (19%) than in Asian and black men. The pathogenesis of AGA is highly variable and remains elusive; however, the presence of dihydrotestosterone (DHT) and degree of genetic predisposition plays a causative role in its development [4, 5]. There are several traditional treatment options available for the treatment of AGA; however, their effectiveness remains limited. Therefore, a safe and effective treatment modality with fewer side effects that can significantly benefit patients with AGA in a dermatology practice setting is highly desirable.

Accumulating studies have indicated that increased levels of DHT and overexpression of the androgen receptor (AR) gene were found to be associated with AGA [6]. Furthermore, 5*α*-reductase present within the dermal papilla plays a crucial role in the intrafollicular conversion of systemic and local testosterone into DHT. DHT binds to the follicular AR, which eventually induces signaling pathways associated with progressive miniaturization and hair loss [7]. Thus, attenuation of levels of DHT is vital for the prevention and treatment of AGA.

Botulinum toxin type A (BTA) is a highly potent neurotoxin that selectively prevents the release of acetylcholine at the neuromuscular junction and is most extensively used in the treatment of a various dermatological disorder [8, 9]. A recent, alternative approach is the injection of BTA into the scalps of patients with AGA. Though a paucity of studies showed that BTA has been tried for AGA management, they had limitations including small sample sizes, unclear evaluation standards, the loss to follow-up of many patients, and lack of combination therapy with other drugs. Therefore, more studies are required to establish the role of BTA in the treatment of AGA.

The present study is aimed at evaluating the safety and effectiveness of BTA on the treatment of AGA.

## 2. Methods

### 2.1. Participants

This study enrolled 63 patients who were presented and diagnosed with AGA at the Department of Dermatology, Hainan Hospital of People's Liberation Army General Hospital, between February 2017 and February 2019. Patients with AGA were randomly divided into BTA and BTA+finasteride (FNS) groups (We have used a simple randomization grouping method, which was to directly randomize the subjects, and generate random numbers by computer for randomization, without any restrictions or interventions or adjustments in advance or in the implementation process). AGA patients in the BTA group (*n* = 30) received BTA (Botox; Allergan, USA) injection, and AGA patients in the BTA+FNS group (*n* = 33) received BTA injection in combination with oral FNS (Propecia; MSD, UK; 1 mg/d). Inclusion criteria were as follows: men aged 18-60 years with a confirmed diagnosis of AGA; AGA diagnosis was evaluated following Norwood Hamilton grade II-IV criteria, patients with no history of treatment with drugs that are known to interfere with BTA within last 6 months; and willingness to provide pictures and follow-up studies. Patients were excluded if presented with severe diseases of internal organs, eyes, or skin; neuromuscular system diseases; inflammation, infection, or unhealed wounds on the skin around the site of injection on the head; systematic treatment with corticosteroids or other immunosuppressants and immunomodulators in the past 3 months; and phobic about treatment with BTA.

### 2.2. Study Design

The study was approved by the Ethics Committee of Hainan Hospital of People's Liberation Army General Hospital. This study conformed to the Declaration of Helsinki, and all participants provided written informed consent. All patients received the treatment for 12 months and were followed up every 3 months, 4 times in total during and after completion of the treatment. Any alteration in hair growth of the head and associated adverse events were recorded during the treatment and at each follow-up visit. Representative photographs of the head were acquired before and after each treatment.

### 2.3. Botulinum Toxin Injection

BTA (100 U/ml, Botox; Allergan, USA) was diluted with 3 ml of 0.9% normal saline. The scalp was sterilized with iodophor, and 30 injection target sites (located in the frontal muscle, temporal muscle, periauricular muscle, and occipital muscle) were marked with gentian violet, and each injection site was 1.5-2 cm apart. BTA at the total injection dose of 100 U/ml was administered intramuscularly using an insulin syringe of 40 U/ml capacities. Participants received BTA injection every 3 months, 4 times in total.

### 2.4. Efficacy Assessment

The efficacy of treatment was evaluated using the hair counts statistics and objective evaluation score of the photographs of the head before and after treatment by dermatologists as the primary outcome and the self-evaluation score of the patients as the secondary outcome.

#### 2.4.1. Hair Counts Statistics

Professional photographic camera (Canon EOS 90d) equipped with a lens (Canon ef-s18-135 mm f/3.5-5.6 is USM) and tripod (to ensure the identical angle and distance for image acquisition) was used for the study. After each treatment, the hairstyle of the patients was kept consistent with that before the treatment. The hair counts in the test before each session and by the end of follow-up were determined according to Canfield's statistical method by an experienced dermatologist [10]. The measurement area of the scalp was identified by the minitattoo, and hair count assessments were performed using a 2 cm^2^ circular template centered over the minitattoo.

#### 2.4.2. Evaluation Score of Head Photos

To assess the improvement in hair growth, two independent dermatologists evaluated the photographic images of the head before each session and by the end of follow-up. Based on the change in hair density in the photographs of the head, the effects of treatment were evaluated using the 4-point scale with 0 defined as poor, 1 as fair, 2 as good, and 3 as excellent. The effective rate was calculated by (good + excellent)/(poor + fair + good + excellent) × 100%.

#### 2.4.3. Self-Assessment Evaluation of Patients

Before and after each treatment, patients were requested to complete a questionnaire. Data on hair loss, dandruff, and scalp oil secretion were also recorded.

### 2.5. Evaluation of Adverse Effects

Data on localized adverse effects including inflammation, erythema, edema, and blisters at the injection site and systemic adverse effects including fever, headache, chest tightness, and nausea were collected.

### 2.6. Statistical Analysis

Quantitative data were expressed as mean ± standard deviation and compared using Student's *t*-test or repeated measures analysis of variance (ANOVA). Qualitative data were expressed as frequency (percentage) and compared using the chi-square test. All data were analyzed using SPSS 17.0. A *P* value of <0.05 was considered statistically significant.

## 3. Results

A total of 63 patients with AGA enrolled to this study. Patients' demographic and clinical characteristics of both groups were summarized in Table 1. There was no significant difference in age, BMI, age hair loss first noticed, and disease durations between BTA and BTA+FNS groups. The Norwood-Hamilton grade and hair counts before treatment also showed no difference between the two groups.

### 3.1. Efficacy

**Figure 1:**
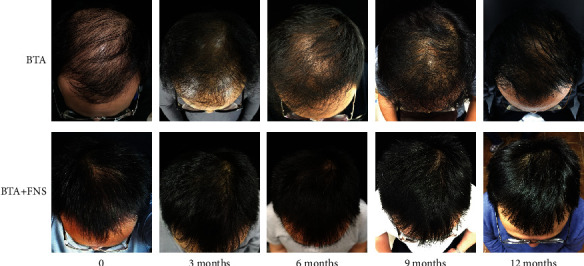
Clinical effectiveness between two groups at different time. BTA: botulinum toxin type A; BTA+FNS: botulinum toxin type A+finasteride.

Hair counts in both groups at all time points were significantly higher compared with those before treatment (all *P* < 0.05) and further increased gradually with prolongation of the treatment time (Table 2). After an overall treatment for 4 times, hair counts in the BTA+FNS group were 234.01 ± 27.35 root/cm^2^ and 218.26 ± 30.59 root/cm^2^ in the BTA group, and the hair counts of both groups were significantly different (*P* < 0.05). However, no significant difference was observed in hair counts between two groups when measured after treatment for once, twice, and three times. Representative photographic images of patients with AGA before and after each treatment were presented in Figure 1. Hair density was significantly augmented, and the target area of hair loss was attenuated after each treatment, suggesting distinctive improvement with BTA and BTA+FNS treatment. The effective rates for BTA and BTA+FNS groups were 73.3% and 84.8% after 4 times treatment, respectively; however, no significant difference in the efficacy of BTA and BTA+FNS on patients with AGA was observed between the two groups (Table 3).

### 3.2. Patient Satisfaction

**Figure 2:**
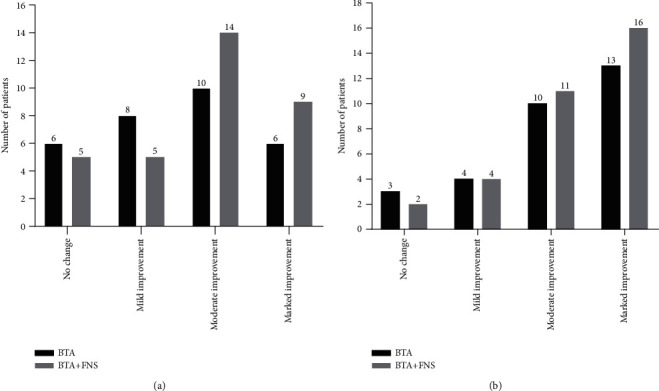
Self-assessment evaluation of patients based on questionnaires. (a) Degree of improvement on symptoms of scalp oil secretion, pruritus, and dandruff. (b) Degree of improvement on reduction in hair loss. BTA: botulinum toxin type A; BTA+FNS: botulinum toxin type A+finasteride.

After treatment, 16 AGA patients in BTA group and 23 patients in BTA+FNS group reported that they exhibited moderate and marked improvement in fewer symptoms of scalp oil secretion, pruritus, and dandruff (Figure 2(a)). Moreover, 23 AGA patients in BTA group and 27 AGA patients in BTA+FNS group experienced a moderate and marked reduction in hair loss as compared with before treatment (Figure 2(b)).

### 3.3. Safety and Tolerability

There were no severe adverse events occurred in all patients. One patient in the BTA group developed headache, possibly related to study medication. Injection site events (pain, erythema, or edema) were developed in 2 patients and 1 patient in the BTA and BTA+FNS group, respectively. A slight breathlessness and nausea associated with BTA injection was reported by 1 patient in BTA+FNS group.

## 4. Discussion

AGA is an androgen-dependent and predominantly genetically determined trait characterized by a chronic and progressive miniaturization of hair follicles [11]. Previous epidemiological studies indicated that men with paternal AGA history is an important risk factor for early onset of AGA in men, while men with a maternal family history were associated with poor prognosis [12, 13]. The whole-genome sequencing and mapping studies have identified several susceptibility loci for AGA; however, the pathogenic genes associated with AGA have not been identified so far [14]. Although the etiology and pathogenesis of AGA remain elusive, recent studies demonstrated that androgen functions as a crucial factor in the pathogenesis of AGA [15]. An increasing number of studies have suggested that 5-alpha reductase converts testosterone to DHT, which binds to hair follicular ARs and leads to the activation of genes that induces miniaturization of the hair follicle and disappearance of hair follicle atrophy, alopecia, and ultimately baldness [16, 17]. Furthermore, the symptoms of AGA could be aggravated by factors such as insufficient blood supply to the scalp, infection in hair follicles, psychological stress or disorder, irregular sleep, and insomnia.

At present, the treatment of AGA mainly includes systemic drugs (such as FNS and spironolactone), external drugs (such as minoxidil), and other alternative methods (hair transplantation, platelet-rich plasma, and low-energy laser) [18]. Notably, combination therapy has been proposed to achieve the best efficacy expediently. However, oral medication requires long-term use and exhibit certain associated side effects, topical medication has limited and inaccurate efficacy, and invasive treatment carries surgical risks [19–21]. And hair loss is associated with considerable psychological and emotional distress and decreased quality of life. Therefore, a safe and effective treatment modality with fewer side effects that can greatly benefit patients with AGA in a dermatology practice setting is highly desirable.

**Figure 3:**
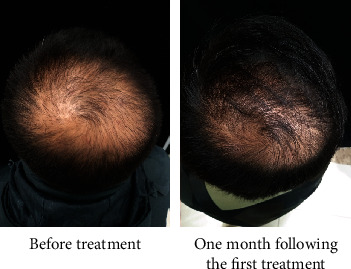
Evaluation of clinical effects on growth and density of hairs one month following the first treatment with BTA.

**Figure 4:**
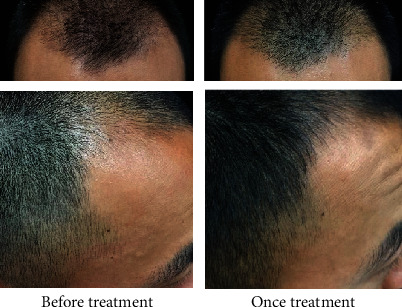
Evaluation of clinical effects on growth and density of hairs at forehead and temples following injection with BTA.

**Figure 5:**
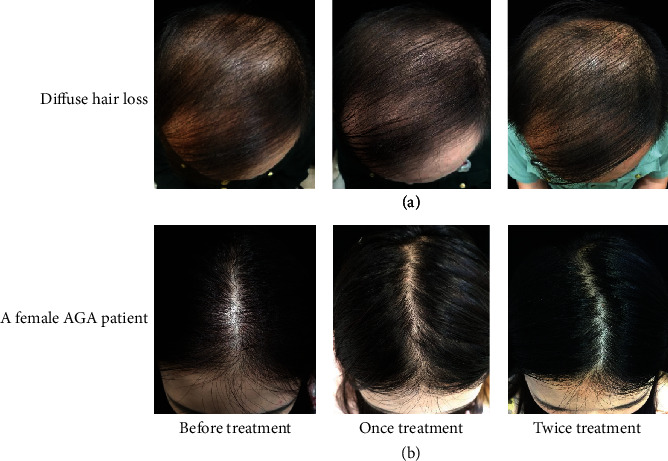
Evaluation of clinical effects from (a) a AGA patient with diffuse hair loss and (b) a female AGA patient on growth and density of hairs following treatment with BTA+FNS.

Botulinum toxin is one of the highly effective neurotoxins produced by the bacterium *Clostridium botulinum*. BTA prevents the release of acetylcholine and many other neurotransmitters at the presynaptic neuromuscular junction [22]. BTA has been extensively used in the dermatology clinic for wrinkle reduction, facial muscle adjustment, hyperhidrosis, correction of masseter hypertrophy, and gastrocnemius hypertrophy. Besides, long-term effects of muscles surrounding the affected scalp (including the frontal, occipital, periauricular, and temporal muscles) tighten the affected scalp, resulting in reduced blood flow to the terminal vessels at the top of the head and forehead, which eventually leads to a hypoxic state in these affected areas [23]. Moreover, DHT is prone to activation under hypoxic conditions, and it is the most crucial factor that terminates hair follicles and causes hair loss. Previous studies suggested that there were hemodynamic abnormalities including microvascular dysfunction and reduced blood flow in the alopecia region of AGA patients [24]. Therefore, improving the scalp blood supply may stimulate hair growth. Possibly, BTA may relax the muscles around the head, increase blood flow and oxygen concentration in the alopecia area, and further inhibit the activation of DHT, ultimately leading to a reduced occurrence of hair loss. Moreover, a high concentration of oxygen can stimulate the hair follicle into the growth phase, resulting in hair regeneration [25–27]. In the present study, we demonstrated that the treatment of the alopecia region with BTA significantly increased hair counts of patients with AGA, and the effect of BTA alone was lower compared to that of BTA combined with FNS. Hair growth and density were also significantly elevated after each treatment as revealed from representative photographs of patients with AGA, and both groups presented a high effective rate. Our data indicated that BTA was effective for the treatment of AGA without noticeable side effects and complications, and BTA combined with FNS exhibited a rapid and better therapeutic effect. Previous studies indicated that an increase in hair growth would only be observed with oral FNS for more than three months, and FNS's influence on hair density was poor on the frontal scalp and least effective bitemporally [28–30]. We found a significant improvement in hair growth and density in several patients one month after the first treatment (Figure 3). Also, compared with FNS treatment, we observed that BTA injection was more effective in regulating the hair density at the forehead and temples in some patients (Figure 4). However, these clinical characteristic findings warrant further investigation. Moreover, the self-assessment of patients for scalp dandruff, pruritus and greasy scalp symptoms were significantly alleviated, and the thickness and texture of the hair were markedly ameliorated one month after the first treatment. We also found that treatment with BTA+FNS was less effective for AGA patients with diffuse hair loss (Figure 5(a)). In addition, we tried to use BTA for a female AGA patient, and our results indicated that BTA may increase growth and density of hairs as revealed by head photographs (Figure 5(b)). However, further clinical studies on a large sample size are needed to validate these findings.

This study still had several limitations. The number of study patients was only 63, which means that additional studies including large populations are required. No evaluation of plasma and scalp DHT levels was assessed in our study. Excluding hair counts, other trichoscopic parameters, including dermoscopic images, hair diameter, and the proportion of miniaturized hairs could be used in monitoring treatment efficacy in the further study.

In conclusion, the findings of the present study demonstrated that BTA is a safe and effective therapy for the management of AGA and BTA combined with FNS presents excellent results. These data provide a novel theoretical basis and therapeutic strategy for the treatment of AGA.

## Figures and Tables

**Table 1:** Demographic and clinical characteristics.

	BTA (*n* = 30)	BTA+FNS (*n* = 33)	*P* value
Age (years)	38.47 ± 10.13	40.06 ± 11.85	0.571
BMI (kg/m^2^)	23.12 ± 4.54	23.89 ± 3.72	0.463
Age hair loss first noticed (years)	31.27 ± 6.35	29.67 ± 8.01	0.386
Disease duration (years)	8.85 ± 7.17	7.83 ± 7.90	0.596
Norwood-Hamilton grade, *n* (%)			
II vertex	9 (30.0)	7 (21.2)	
III	13 (43.3)	15 (45.4)	
IV	8 (26.7)	11 (33.3)	0.696
Hair counts before treatment (root/cm^2^)	180.57 ± 26.53	178.21 ± 24.33	0.715

BTA: botulinum toxin type A; BTA+FNS: botulinum toxin type A+finasteride.

**Table 2:** Evaluation of hair counts between two groups at different time.

	BTA (*n* = 30)	BTA+FNS (*n* = 33)	*P* value
Hair counts at different time (root/cm^2^)			
0 (before treatment)	180.57 ± 26.53	178.21 ± 24.33	0.715
3 months (once treatment)	196.03 ± 32.89	205.78 ± 28.50	0.212
6 months (twice treatment)	208.04 ± 27.00	220.44 ± 27.06	0.074
9 months (three times treatment)	214.83 ± 31.17	228.31 ± 30.99	0.091
12 months (four times treatment)	218.26 ± 30.59	234.01 ± 27.35	0.035
^a^ *P* value	0.015	<0.001	
^b^ *P* value	<0.001	<0.001	
^c^ *P* value	<0.001	<0.001	
^d^ *P* value	<0.001	<0.001	

BTA: botulinum toxin type A; BTA+FNS: botulinum toxin type A+finasteride; ^a^*P* value was derived from comparisons between 0 and 3 months; ^b^*P* value was derived from comparisons between 0 and 6 months; ^c^*P* value was derived from comparisons between 0 and 9 months; ^d^*P* value was derived from comparisons between 0 and 12 months.

**Table 3:** Evaluation score and effective rate between two groups.

	BTA (*n* = 30)	BTA+FNS (*n* = 33)	*P* value
Evaluation score after 4 times treatment, *n* (%)			
0	3 (10.0)	2 (6.1)	
1	5 (16.7)	3 (9.1)	
2	9 (30.0)	10 (30.3)	
3	13 (43.3)	18 (54.5)	
Effective rate (%)	73.3	84.8	0.259

BTA: botulinum toxin type A; BTA+FNS: botulinum toxin type A+finasteride.

## Data Availability

The data used to support the findings of this study are included in this published paper and can be available from the corresponding author upon request.
